# Extracellular DNA concentrations in various aetiologies of acute kidney injury

**DOI:** 10.1038/s41598-022-21248-7

**Published:** 2022-10-07

**Authors:** Alexandra Gaál Kovalčíková, Ľubica Janovičová, Július Hodosy, Janka Bábíčková, Diana Vavrincová-Yaghi, Peter Vavrinec, Peter Boor, Ľudmila Podracká, Katarína Šebeková, Peter Celec, Ľubomíra Tóthová

**Affiliations:** 1 Institute of Molecular Biomedicine, Faculty of Medicine, Comenius University, Sasinkova 4, 811 08 Bratislava, Slovakia; 2 Department of Paediatrics, Faculty of Medicine, National Institute of Children’s Diseases, Comenius University in Bratislava, Bratislava, Slovakia; 3 Emergency Department Ruzinov, University Hospital Bratislava, Bratislava, Slovakia; 4 Department of Clinical Medicine, University of Bergen, Bergen, Norway; 5 Department of Pharmacology and Toxicology, Faculty of Pharmacy, Comenius University, Bratislava, Slovakia; 6 Department of Nephrology, Institute of Pathology, University Clinic of the RWTH Aachen, Aachen, Germany; 7 Institute of Pathophysiology, Faculty of Medicine, Comenius University, Bratislava, Slovakia

**Keywords:** Biomarkers, Nephrology

## Abstract

Extracellular DNA (ecDNA) in plasma is a non-specific biomarker of tissue damage. Urinary ecDNA, especially of mitochondrial origin, is a potential non-invasive biomarker of kidney damage. Despite prominent tissue damage, ecDNA has not yet been comprehensively analysed in acute kidney injury (AKI). We analysed different fractions of ecDNA, i.e. total, nuclear and mitochondrial, in plasma and urine of children, and different animal models of AKI. We also analysed the activity of the deoxyribonuclease (DNase), which is contributes to the degradation of ecDNA. Patients with AKI had higher total and nuclear ecDNA in both, plasma and urine (sixfold and 12-fold in plasma, and 800-fold in urine, respectively), with no difference in mitochondrial ecDNA. This was mainly found for patients with AKI due to tubulointerstitial nephritis and atypical haemolytic uremic syndrome. Increased plasma ecDNA was also found in animal models of AKI, including adenine nephropathy (fivefold), haemolytic uremic syndrome (fourfold), and ischemia–reperfusion injury (1.5-fold). Total urinary ecDNA was higher in adenine nephropathy and ischemia–reperfusion injury (1300-fold and twofold, respectively). DNase activity in urine was significantly lower in all animal models of AKI in comparison to controls. In conclusion, plasma total and nuclear ecDNA and urinary total ecDNA is increased in patients and animals with particular entities of AKI, suggesting a mechanism-dependent release of ecDNA during AKI. Further studies should focus on the dynamics of ecDNA and its potential role in the pathogenesis of AKI.

## Introduction

Acute kidney injury (AKI) is a life-threatening syndrome with high prevalence and complex aetiology^1^. The current gold standard diagnostic methods, such as serum creatinine, cystatin C, and eGFR are unreliable to detect early phases of AKI. Despite the progress in identifying novel biomarkers of AKI, such as kidney injury molecule-1 (KIM-1), neutrophil gelatinase-associated lipocalin-2 (NGAL), and interleukin-18 (IL-18), their use in clinical practice is limited due to lack of cut off values as well as validation studies in larger population^2,3^. Late therapeutic intervention in AKI increases the risk of progression to end-stage renal disease and irreversible kidney damage^4,5^. Thus, there is urge for early non-invasive and sensitive biomarkers for diagnostics of AKI, that could also serve for the prediction of AKI development in critically ill patients^6,7^.

Extracellular DNA (ecDNA)—a non-cellular DNA present in the majority of body fluids including plasma and urine—is a non-specific marker of tissue damage, which is present in the form of fragments of genomic or mitochondrial DNA (mtDNA). Unlike DNA fragments of nuclear origin, i.e. nuclear DNA (ncDNA), which is protected against degradation in nucleosomes, mtDNA is protein-free and thereby more fragmented^8^. Concentrations of ecDNA increase during cell death (e.g., apoptosis, necrosis, or NETosis)^9,10^. The ecDNA is recognized by immune cells and induces inflammation^11^. Activated immune cells might further induce cell damage and release of DNA, and contribute to a *circulus vitiosus* further promoting organ injury^12^.

Interest in ecDNA has increased over the past years. Several studies described the potential role of ecDNA in the pathogenesis of chronic kidney disease^13–15^ as well as AKI^16,17^. Regarding AKI, septic patients who developed AKI displayed higher concentrations of ecDNA compared to those without AKI^16^. Likewise, ecDNA has accurate predictive value for the development of AKI in patients after cardiac surgery. In patients who developed AKI during the first 5 post-operative days, ecDNA was higher immediately after surgery^17^. Levels of plasma and urinary ecDNA seem to be comprehensive markers to monitor allograft function following kidney transplantation. Increased concentrations of donor-derived ecDNA can detect asymptomatic injury to allograft, and predict acute and chronic graft rejection without the need for biopsy^18,19^. Furthermore, ecDNA appears to be involved in complications following peritoneal dialysis and haemodialysis, its levels correlate with the clinical status of patients and with inflammatory markers^20–22^.

We have previously shown that mice with experimental bilateral ureteral obstruction or rhabdomyolysis have higher concentrations of plasma ecDNA compared to controls^23^. Increased concentrations of ecDNA in plasma and urine were also observed in models of ischemia–reperfusion injury (IRI) and rhabdomyolysis that were associated with the release of ecDNA from necrotic tubular epithelial cells and activated granulocytes^24–26^.

Previous studies showed that ecDNA concentrations are elevated in patients with AKI. However, these studies neither differentiated the ecDNA fractions, nor determined DNase activity either in plasma or urine in children with various aetiologies of AKI. Different AKI models have not been studied either. We hypothesized that concentrations of ecDNA vary based on the origin of AKI. Therefore, our cross-sectional study aimed to assess concentrations of plasma and urinary ecDNA in paediatrics patients with different AKI origins and three animal models of AKI. Analysis in children enable minimizing comorbidities potentially affecting concentrations of ecDNA as well as DNase activity.

## Methods

### Clinical study

The cross-sectional clinical study was performed in compliance with the Declaration of Helsinki. Patients and healthy controls were included from the study titled “Non-invasive yet unused markers of renal function: problems, causes and opportunities” that was approved by the Ethics committee of the National Institute for Children’s Diseases in May 2019. Informed consent was obtained from legal guardians of children participating in this study.

Forty-two consecutive paediatric patients diagnosed with AKI admitted to the Paediatric Nephrology Clinic of the National Institute of Children’s Diseases, Faculty of Medicine, Comenius University in Bratislava, Slovakia from May 2019 to March 2021 were included. Paediatric-modified RIFLE (Risk for renal dysfunction, Injury to the kidney, Failure of kidney function, Loss of kidney function, and End-stage renal disease) criteria were used as a standard to diagnose AKI^27,28^. Estimated glomerular filtration rate (eGFR) was calculated according to modified Schwartz formula^29^. Repeated samplings, haemolysed or contaminated samples were excluded from analyses. Finally, 28 patients (range between 1 and 18 years) were included. Clinical data of patients and respective controls are summarized in Supplementary Table S1. Twenty-seven age- and sex-matched healthy children (range 3–18 years) with no prior history of kidney disease were recruited from children attending regular check-ups at the practice of general paediatrician at the same Institute in the same timeframe.

All samples were collected in the morning hours between 6 and 8 am after overnight fasting. Venous blood was collected from the cubital vein, into K_3_EDTA and lithium-heparin (BD Vacutainer Plastic Tube, Becton Dickinson, Czech Republic). Spot urine was collected into sterile Falcone tubes (Sarstedt, Nümbrecht, Germany).

### Animal experiments

This study was conducted according to relevant national legislation for the use of animals in research and was approved by the Ethics Committee of the Institute of Pathophysiology, Faculty of Medicine, Comenius University, Bratislava, Slovakia, and the State Veterinary and Food Administration of the Slovak Republic. The study was carried out in compliance with the ARRIVE (Animal Research: Reporting of In Vivo Experiments**)** guidelines. All animals were housed in standard cages with controlled 12/12 h light/dark cycle, constant temperature (22 ± 2 °C), and humidity (45–65%) with ad libitum access to water and chow.

### Adenine nephropathy (AN)

Male C57BL/6 mice (10–24 weeks old, Anlab, Prague, Czech Republic) were randomized into 2 groups. To induce AN (n = 15), adenine hydrochloride hydrate dissolved in saline (100 mg/kg, Sigma Aldrich, Münich, Germany) was applied daily by intraperitoneal injection for 3 weeks^30^. Age- and sex- matched control mice (n = 15) received saline. After 3 weeks, mice were placed into individual metabolic cages to collect urine. Under anaesthesia (ketamine and xylazine,100 mg/kg and 10 mg/kg, i.p., in ratio 3:1), blood was collected from inferior vena cava into K_3_EDTA and lithium-heparin tubes (Sarstedt, Nümbrecht, Germany) and animals were sacrificed by cervical dislocation.

### Haemolytic uremic syndrome (HUS)

To induce HUS, adult male C57BL/6 mice (n = 16, 10–24 weeks old, Anlab, Prague, Czech Republic) were intraperitoneally injected with a single dose of Shiga toxin (STX2, Tufts University, Boston, Massachusetts, 30 µg/kg dissolved in 100 µl of saline)^31^. Mice in the control group (n = 8) received intraperitoneal injection of 100 µl of saline. To mitigate dehydration, both groups received intraperitoneal injection of 500 µl of saline twice a day. After 48 h, urine and blood were collected.

### Ischemia–reperfusion injury (IRI)

Male Wistar rats (10–12 weeks old, Anlab, Prague, Czech Republic) were randomized into 2 groups. To induce IRI, rats (n = 28) were anesthetised in the same manner as described above. Surgery was performed as described elsewhere^32^. Briefly, animals were anesthetized, an abdominal incision was performed and kidneys were exposed. The ischemic injury was induced by the application of micro-clamps on renal pedicles for 30 min. Body temperature was maintained by a heating pad. After the ischemic phase, clamps were released and reperfusion was confirmed visually. The muscular layer was closed with an absorbable suture (Chirlac 4–0, Chirmax, Praha, Czech Republic). The skin was closed using clips (Sureline Skin Stapler Accessories, 35 W, Patterson Companies, Staffordshire, England, United Kingdom). Sham animals (n = 18) underwent the same procedure except that the clamps were not applied. After 48 h, urine and blood samples were collected.

### Blood and urine sample processing

Blood and urine samples were immediately centrifuged at 1600 g for 10 min. Supernatants were divided into 2 aliquots. One aliquot used for the isolation of ecDNA was further centrifuged at 16 000 g for 10 min to remove apoptotic bodies and subsequently stored at − 20 °C until DNA isolation. The second aliquot was stored at − 20 °C until biochemical analysis.

### Biochemical analysis (kidney function assessment)

Plasma creatinine and urea nitrogen were measured using commercially available spectrophotometric assay according to protocols recommended by the manufacturer (Creatinine Serum Low Sample Volume; Urea Nitrogen Colorimetric Detection Kit, Arbor Assays, Ann Arbor, USA). For creatinine measurement, 15 µl of plasma samples were used. The absorbance was measured at 490 nm after 1st and 30th minutes, 1st minute serving as the baseline. Sensitivity for creatinine was determined as 7.16 µmol/l. For the measurement of urea, 10 µl of plasma samples were 10-times diluted with distilled water and mixed with Colour Reagent A and Colour Reagent B. Absorbance was measured at 450 nm with the sensitivity of 0.01 mmol/l.

Myeloperoxidase (MPO) in plasma and urine samples was assessed using commercial ELISA kit as recommended by manufacturer protocol (Human Myeloperoxidase DuoSet Elisa Kit, R&D Systems, Minneapolis, USA). For plasma measurement, samples were diluted 100-times with Reagent Diluent (1% BSA in PBS). Urine samples were diluted 5-times in the same way. Absorbance was measured at 450 nm.

Creatinine in urine samples was measured using the Jaffé method^33^. Briefly, 10 µl of samples and standards were mixed with 200 µl of freshly prepared working solution (0.2 M NaOH and 25 mM picric acid, 5:1 ratio). Absorbance was measured at 492 nm after the 1st and 6th minutes. In calculation, initial absorbance was subtracted from the second absorbance. Sensitivity was determined as 16 µmol/l.

### ecDNA isolation

DNA was isolated from 200 µl of plasma or urine using a commercial kit (QIAamp DNA Mini Kit, Qiagen, Hilden, Germany) according to the manufacturer’s protocol. Fifty microliters of ultrapure water were used for the elution of DNA. DNA samples were stored at − 20 °C for further analysis.

### ecDNA quantification

The concentration of total ecDNA was quantified using a fluorometric method according to the manufacturer’s protocol (Qubit Fluorometer and Qubit dsDNA HS Assay Kit (Invitrogen, Carlsbad, CA, USA). NcDNA and mtDNA in isolates were quantified using real-time polymerase chain reaction (real-time PCR) on the MasterCycler RealPlex^4^ (Eppendorf, Hamburg, Germany) using the SsoAdvanced Universal SYBR Green Supermix (Bio-Rad, Hercules, CA, USA). For isolates from clinical samples, human beta-globin gene (F: 5′- GCT TCT GAC ACA ACT GTG TTC-3′, R: 5′- CAC CAA CTT CAT CCA CGT TCA-3′) and primers targeting D-loop (F: 5′- CAT AAA AAC CCA ATC CAC ATC A-3′, R: 5′- GAG GGG TGG CTT TGG AGT-3′) amplification were used to quantify ncDNA and mtDNA, respectively.

To quantify ncDNA in animal samples, primers were designed for the amplification of mouse beta-2-microglobulin gene (F: 5′-TGT CAG ATA TGT CCT TCA GCA AGG-3′, R: 5′-TGC TTA ACT CTG CAG GCG TAT G-3′) and rat GAPDH gene (F: 5′-GAA ATC CCC TGG AGC TCT GT-3′, R: 5′-CTG GCA CCA GAT GAA ATG TG-3′). Mouse and rat cytochrome B genes were used for mtDNA quantification (mice: F: 5′-CCC AGC TAC TAC CAT CAT TCA AGT-3′, R: 5′-GAT GGT TTG GGA GAT TGG TTG ATG T-3′; rats: F: 5′-CCT CCC ATT CAT TAT CGC CGC CCT TGC-3′, R: 5′-GTC TGG GTC TCC TAG TAG GTC TGG GAA-3′).

### DNase activity measurement

Single radial enzyme-diffusion (SRED) with GoodView Nucleic Acid Stain (SBS Genetech, Beijing, China) was used for the measurement of DNase activity in lithium-heparin plasma and urine. One microliter of each sample was analysed in 1% agarose gel (2 mM MgCl2, 2 mM CaCl2, 20 mM Tris–HCl pH 7.5, 0.035 mg/mL DNA isolated from porcine livers). The calibration curve was made of DNase I standard of known DNase activity, with serial two-fold dilutions in the RDD buffer presented in the set (RNase-free DNase set, Qiagen, Hilden, Germany). After overnight incubation of gel (16–20 h, in the dark) at 37 °C, the gel was visualised in iBOX (Vision works LP Analysis Software, UVP, Upland, CA, USA). The circle diameters were measured using ImageJ software (NIH, Bethesda, Maryland, MD, USA). DNase activity calculation was based on comparison to the calibration curve. The activity was expressed in Kunitz units (K.u.) per mL of sample.

### Statistical analysis

GraphPad Prism 6.01 (GraphPad Software Inc, La Jolla, USA) was used for statistical analysis. D'Agostino-Pearson omnibus test was used to test the normality of data distribution. Outliers were detected using the Grubbs test. To compare normally distributed data, a two-sided Student’s t-test was used. Not normally distributed data were analysed using the Mann–Whitney U-test. Data with high variability were log-transformed. To compare concentrations of ecDNA in patients with different aetiopathologies of AKI, the Kruskal–Wallis test and subsequently Dunn’s multiple comparison test was used. Relations between kidney functions and clinical data, respectively, and ecDNA were tested by Spearman’s rank-order correlation coefficient. All data are expressed as mean ± SD. A *p*-value less than 0.05 was considered statistically significant.

## Results

### Clinical study

**Figure 1:**
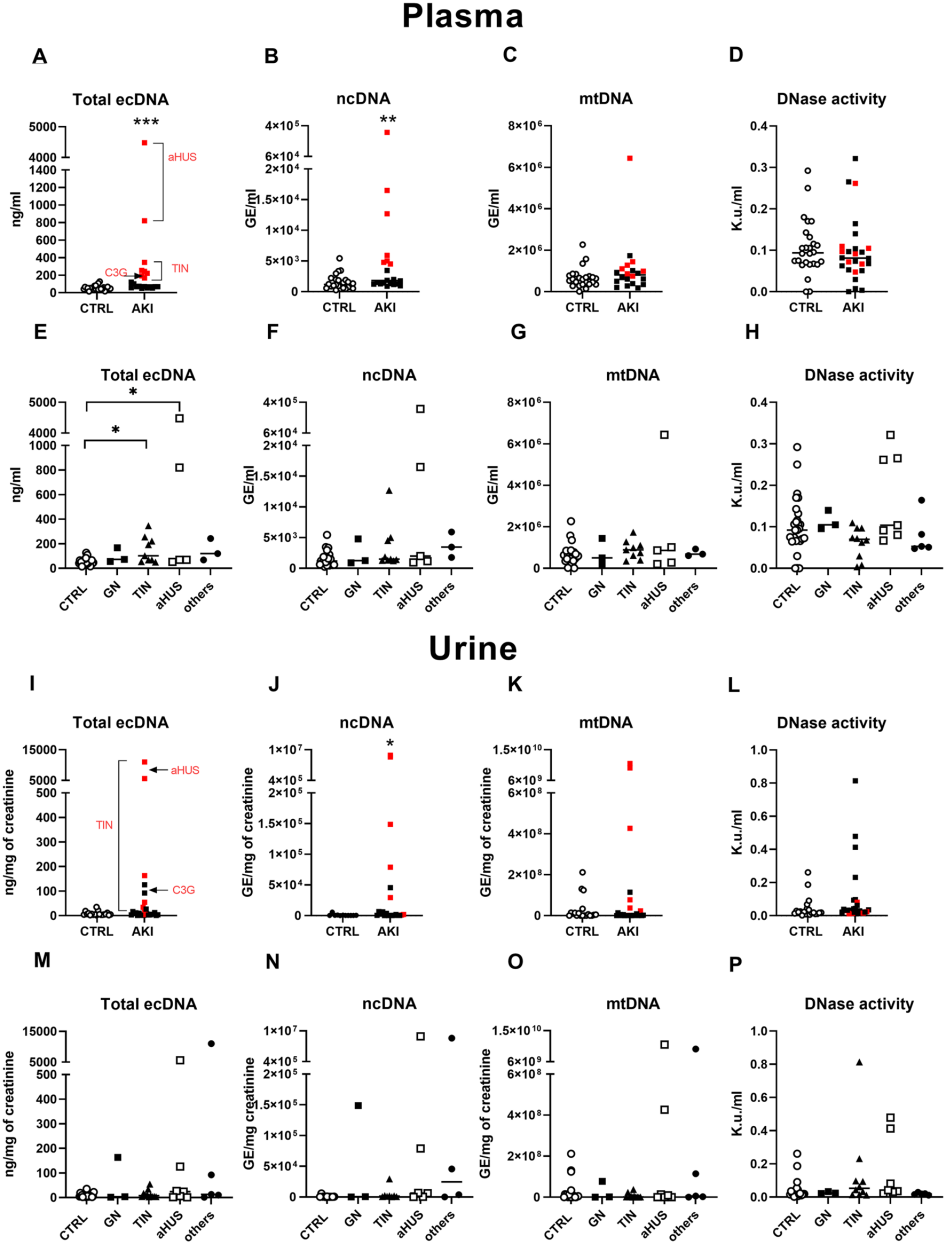
Concentrations of plasma and urinary ecDNA and DNase activity in children with AKI and healthy controls. Plasma (**A**) total ecDNA, (**B**) ncDNA, (**C**) mtDNA, (**D**) activity of DNase in children with AKI regardless the aetiology and in healthy controls. Plasma (**E**) total ecDNA, (**F**) ncDNA, (**G**) mtDNA, (**H**) activity of DNase in plasma of children with different aetiologies (subgroups) of AKI and healthy children. Urinary (**I**) total ecDNA, (**J**) ncDNA, (**K**) mtDNA, (**L**) activity of DNase in all AKI patients regardless the aetiology and in healthy individuals. Urinary (**M**) total ecDNA, (**N**) ncDNA, (**O**) mtDNA, (**P**) activity of DNase in children with different aetiologies (subgroups) of AKI and healthy children. Results are presented by individual values plots with median. * denotes *p* ˂ 0.05, ** denotes *p* ˂ 0.01, *** denotes *p* ˂ 0.001 in comparison to healthy controls. *DNase* deoxyribonuclease, *ecDNA* extracellular DNA, *mtDNA* mitochondrial DNA, *ncDNA* nuclear DNA.

The total plasma ecDNA and ncDNA concentrations were significantly higher in children with AKI compared to healthy children (ecDNA: 55 vs. 379 ng/ml, U = 67, *p* < 0.001; ncDNA: 1549 vs. 19,866 GE/ml, U = 131, *p* < 0.01). Children with AKI had similar concentrations of mtDNA as healthy controls (U = 178, *p* = ns). DNase activity did not differ significantly between children with AKI and healthy children (U = 224, *p* = ns) (Fig. 1A–D).

After subclassification of AKI patients according to aetiology (see Supplementary Table S1), the total plasma ecDNA differed significantly (H = 18.47, *p* < 0.001) in patients with tubulointerstitial nephritis (TIN) and atypical HUS (aHUS) when compared to healthy children (TIN: z = 3.24, *p* < 0.05; HUS: z = 2.74, *p* < 0.05). However, plasma ncDNA and mtDNA did not differ within AKI patients (ncDNA: H = 8.06, *p* = ns; mtDNA: H = 3.74, *p* = ns). DNase activity did not differ within AKI patients (H = 7.60, *p* = ns) (Fig. 1E–H).

Regardless of the aetiology, urinary concentrations of total ecDNA and mtDNA did not differ between AKI patients and controls (ecDNA: U = 220, *p* = ns; mtDNA: U = 231 *p* = ns), while ncDNA levels were 800-fold higher in the AKI group compared to the control group (917 vs. 777,766 ng/ml, U = 57, *p* < 0.05). Children with AKI had twofold higher DNase activity, however, this difference was not statistically significant (U = 164.5, *p* = 0.06) (Fig. 1I–L).

There was no difference in urinary total ecDNA, ncDNA, and mtDNA within AKI patients subgroup (total ecDNA: H = 2.92, *p* = ns; ncDNA: H = 6.11, *p* = ns; mtDNA: H = 1.44, *p* = ns). Also, there were no significant differences in DNase activity within different aetiologies of AKI (H = 8.95, *p* = ns) (Fig. 1M–P).

Patients with AKI displayed significantly higher concentration of MPO in plasma (85,020 vs. 166,645 pg/ml, U = 165.5, *p* < 0.05) and in urine (105 vs. 2322 pg/ml, U = 44, *p* < 0.001) in comparison to healthy controls. After division of AKI patients into subgroups according to aetiology, we did not find any difference in plasma MPO (H = 6.8, *p* = ns). Urinary MPO differed significantly within different aetiologies of AKI (H = 13.92, *p* < 0.01). Significantly higher concentrations of urinary MPO were found in patients with HUS compared to healthy controls (z = 2.84, *p* < 0.05) together with TIN patients showing similar trend (z = 2.27, *p* = 0.09, Supplementary Fig. S1).

### Relation between kidney functions and ecDNA

In the whole group of participants, Spearman’s analysis revealed an inverse correlation between estimated glomerular filtration rate (eGFR) and concentrations of total ecDNA and ncDNA in plasma (r = − 0.47, *p* < 0.001; r = − 0.44, *p* < 0.01, respectively). Moreover, a trend to the inverse relation between eGFR and plasma mtDNA was observed (r = − 0.29, *p* = 0.06). Likewise, urinary ncDNA negatively correlated with eGFR (r = − 0.47, *p* < 0.01) together with total urinary ecDNA showing similar trend (r = − 0.27, *p* = 0.06; Supplementary Table S2).

### Relation between clinical parameters and ecDNA

In patients with different aetiologies, we did not reveal any relations between clinical parameters and concentrations of ecDNA and its fractions. In patients with glomerulonephritis (GN), ecDNA concentrations showed no association with proteinuria. Similarly, in patients with aHUS there was no relation between concentration of lactate dehydrogenase (LDH) and ecDNA in plasma and urine. In patients with TIN or “Others”, Spearman’s rank-order test revealed no association between eGFR and ecDNA (Supplementary Table S3).

### Experimental study

**Figure 2:**
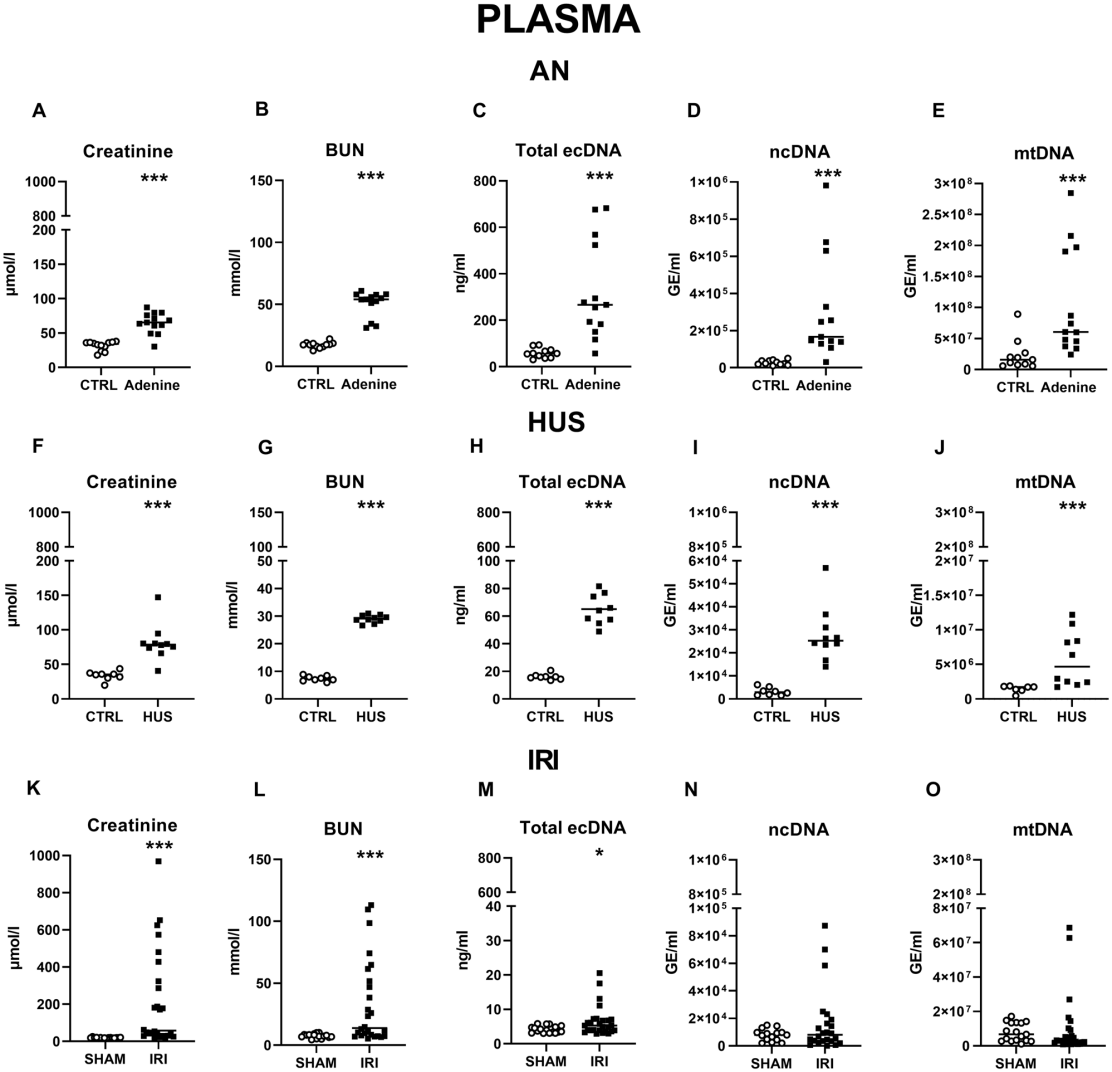
Kidney functions, concentrations plasma ecDNA and its subtypes in different animal models of AKI. Plasma (**A**) creatinine, (**B**) BUN, (**C**) total ecDNA, (**D**) ncDNA, (**E**) mtDNA in mice with AN and control animals. Plasma (**F**) creatinine, (**G**) BUN, (**H**) total ecDNA, (**I**) ncDNA, (**J**) mtDNA in mice with HUS and healthy controls. Plasma (**K**) creatinine, (**L**) BUN, (**M**) total ecDNA, (**N**) ncDNA, (**O**) mtDNA in IRI rats and sham animals. Results are presented by individual values plots with median. * denotes *p* ˂ 0.05, *** denotes *p* ˂ 0.001 in comparison to healthy controls or sham animals, respectively. *AN* adenine nephropathy, *BUN* blood urea nitrogen, *ecDNA* extracellular DNA, *HUS* haemolytic uremic syndrome, *IRI* ischemia–reperfusion injury, *mtDNA* mitochondrial DNA, *ncDNA* nuclear DNA.

To verify the induction of kidney injury in animal models, plasma creatinine and urea were measured. In all models, creatinine and urea were significantly higher in mice with induced kidney injury when compared to control mice (Fig. 2).

### Plasma ecDNA in animal models

In the AN model, significantly higher concentrations of total plasma ecDNA (59 vs. 327 ng/ml, t_22_ = 4.13, *p* < 0.001) were measured in the experimental group compared to the control group. Concentrations of plasma ncDNA were tenfold higher (U = 4, *p* < 0.001) and mtDNA concentrations were fourfold higher in the AN group (U = 13.5, *p* < 0.001) (Fig. 2C–E).

In a model of HUS, a fourfold higher concentration of total plasma ecDNA was observed compared to controls (U = 0, *p* < 0.001). Mice with HUS displayed significantly higher levels of ncDNA and mtDNA in comparison to controls (ncDNA: ninefold higher, U = 0, *p* < 0.001; mtDNA: fourfold higher, U = 3, *p* < 0.001) (Fig. 2H–J).

In the IRI model compared to the sham group, significantly higher concentrations of total plasma ecDNA (1.5-fold higher, U = 138, *p* < 0.05) were observed. There was no significant difference between sham and experimental groups in plasma ncDNA (U = 167.5, *p* = ns) and mtDNA (U = 163.5, *p* = ns) (Fig. 2M–O).

### Urinary ecDNA in animal models

**Figure 3:**
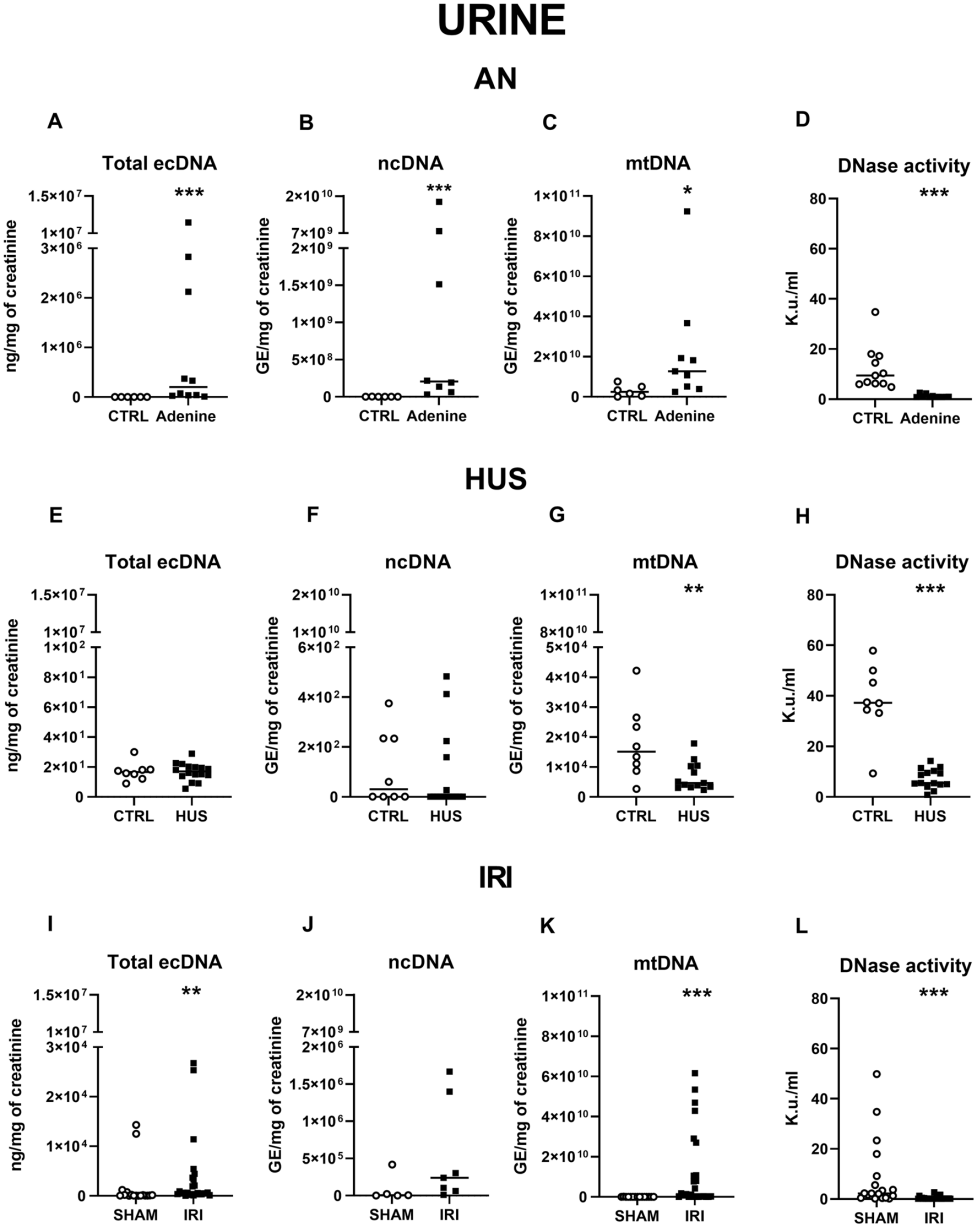
Concentrations of urinary ecDNA, its subtypes and DNase activity in different animal models of AKI. Urinary (**A**) total ecDNA, (**B**) ncDNA, (**C**) mtDNA, (**D**) Dnase activity in AN and healthy mice. Urinary (**E**) total ecDNA, (**F**) ncDNA, (**G**) mtDNA, (**H**) DNase activity in animals with HUS and controls. Urinary (**I**) total ecDNA, (**J**) ncDNA, (**K**) mtDNA, (**L**) DNase activity in IRI rats and sham animals. Results are presented by individual values plots with median. * denotes *p* ˂ 0.05, ** denotes *p* ˂ 0.01, *** denotes *p* ˂ 0.001 in comparison to healthy controls or sham animals, respectively. *AN* adenine nephropathy, *DNase* deoxyribonuclease, *ecDNA* extracellular DNA, *HUS* haemolytic uremic syndrome, *IRI* ischemia–reperfusion injury, *mtDNA* mitochondrial DNA, *ncDNA* nuclear DNA.

In animals with AN, total urinary ecDNA was significantly higher compared to the control group (1 262 vs. 1 728 590 ng/mg of creatinine, U = 0, *p* < 0.001). Correspondingly, urinary ncDNA (488 792 vs. 3 482 749 184 GE/mg of creatinine, U = 0, *p* < 0.001) and mtDNA levels (2 997 999 782 vs. 22 380 798 243 GE/mg of creatinine, U = 6, *p* < 0.05) were significantly higher. (Fig. 3A–D).

Animals with HUS did not differ from healthy mice in the concentration of total urinary ecDNA (t_22_ = 0.09, *p* = ns) or that of ncDNA (U = 52, *p* = ns), while urinary mtDNA was significantly lower threefold in animals with HUS compared to controls (t_19_ = 3.0, *p* < 0.01). Mice with HUS displayed fivefold lower urinary DNase activity compared to control mice (U = 5.5, *p* < 0.001) (Fig. 3E–H).

In IRI, the total urinary ecDNA was twofold higher (U = 105, *p* < 0.01) compared to the sham group. While urinary ncDNA did not differ between IRI and sham groups (U = 6, *p* = ns), those of mtDNA were significantly higher in animals undergoing IRI (11 283 097 vs. 12 330 887 802 GE/mg of creatinine, U = 15, *p* < 0.001). Animals with IRI had lower DNase activity of urine compared to sham animals (23-fold, U = 51, *p* < 0.001) (Fig. 3I–L).

### Relation between kidney functions and ecDNA

In AN, Spearman’s test showed inverse correlation between GFR and ecDNA and its fractions in plasma (r = − 0.75, *p* < 0.001 for ecDNA; r = − 0.68, *p* < 0.001 for ncDNA; r = − 0.62, *p* < 0.01 for mtDNA) as well as in urine (r = − 0.82, *p* < 0.001 for ecDNA; r = − 0.84, *p* < 0.001 for ncDNA; r = − 0.79, *p* < 0.001 for mtDNA).

In HUS, plasma total ecDNA and ncDNA negatively correlated with GFR (r = − 0.85, *p* < 0.001 for ecDNA; r = − 0.78, *p* < 0.001 for ncDNA) with mtDNA showing similar trend (r = − 0.45, *p* = 0.06). No significant relation between GFR and urinary ecDNA was found.

In IRI, GFR negatively correlated with total ecDNA in plasma (r = − 0.31, *p* < 0.05). In urine, GFR inversely correlated with all ecDNA fractions (r = − 0.59, *p* < 0.001 for ecDNA; r = − 0.86, *p* < 0.001 for ncDNA; r = − 0.60, *p* < 0.001 for mtDNA) (Supplementary Table S4).

## Discussion

To our knowledge, this is the first study aiming to characterize different ecDNA fractions in plasma and urine of children with AKI of various aetiology as well as in three different animal models. Results showed that the rise in plasma and urinary ecDNA is cause-specific, i.e. increased in patients with aHUS and TIN, but not in GN. In animals, an increase in plasma and urinary ecDNA was accompanied by decreased urinary DNase activity.

In children, the majority of an acute HUS is generally manifested in genetically predisposed children after the triggering event^34^. Pathophysiologically, HUS is thrombotic microangiopathy manifested in the kidneys where endothelial cells are the main target of the injury^35^. ecDNA is involved in the generation of thrombi by different pathways, including platelet adhesion to DNA fibers, promoting fibrin deposition, and thrombi stabilization^36,37^. EcDNA bonded histones also contribute to thrombogenesis—by induction of platelet aggregation and stimulation of thrombin production, further promoting organ damage with ecDNA release^38–40^. Elevation of circulating ecDNA in our children with aHUS is in line with previously published studies^41,42^. However, regarding total and ncDNA, only a trend towards an increase was observed. This might point towards more complex mechanisms. In the animal model of HUS, all fractions of ecDNA were higher compared to control animals. Unlike in humans, the pathophysiology of HUS in rodents is mainly driven by injury to the tubular epithelium of the collecting ducts. This different site of injury of aHUS in humans and experimental HUS might be reflected by discrepancies of ecDNA dissimilarities in humans and mice^43^.

In humans, the most common causes of TIN are hypersensitivity reactions following drug administration or systemic inflammation^44^. The pathophysiology of TIN involves infiltration of monocytic inflammatory cells, mostly T-lymphocytes, and fewer plasma cells and macrophages. In these patients, the most likely source of ecDNA are injured tubular cells releasing their content caused by inflammatory infiltrates. Moreover, resulting inflammation itself further promotes a rise in ecDNA^45^. On the other hand, necroptosis might be involved in direct drug toxicity to tubular cells during TIN. The released ecDNA activates immune system extending inflammation and release of additional ecDNA in turn. This might be an alternative and/or supplemental source of ecDNA, particularly in the urine^45^.

The animal model of AKI induced by ischemia–reperfusion injury is characterized by injury to proximal tubular cells^46^. Total plasma ecDNA was higher with ncDNA and mtDNA showed similar trends. This finding is in line with previous studies published by our group^23^, as well as others^47^. Interestingly, total ecDNA and mtDNA were significantly higher in the urine. Whether this is a result of the prominent cell shedding to the tubular lumen with subsequent presence in the urine is currently unclear^48,49^.

ecDNA and NETs are characteristic features of several different types of glomerulonephritides, while histones released by NETs are the main contributors to glomerular injury^50,51^. In the present study, neither plasma nor urinary ecDNA differed between GN patient subgroup and healthy controls. A recent study showed that NETosis is more frequent in other types of GN, such as an antineutrophil cytoplasmic antibody (ANCA)-associated vasculitis, or lupus nephritis^52^. In our study, children suffered mainly from IgA GN and complement-3 glomerulopathy, what probably underlies the lack of increase of ecDNA in this subgroup of AKI children. Nevertheless, the number of patients in GN subgroup of AKI did not allow thorough analysis.

In contrast to our GN patients, all measured fractions of ecDNA were significantly higher both in plasma and urine in AN. In this murine model of AKI, the injury originates in the tubulointerstitium, thus, it might be possible that increased ecDNA correlates with tubulointerstitial injury, since it is released directly from damaged cells. Nevertheless, further clarification of this hypothesis is warranted by other studies.

DNase activity in urine and kidneys is slightly higher than in the majority of other mouse tissues^53^. In healthy mice, urinary DNA correlates with kidney DNase activity, but there is no correlation between urinary and serum DNase activity. This suggests that the kidney is the main DNase source in the urine^53,54^. On contrary, several other studies demonstrated that also other tissues such as pancreas could contribute^55^. Nevertheless, the source of urinary nuclease activity remains unclear and warrants further research.

During IRI, an activation of DNase I has been observed in the kidney^56^. Contrastingly, in the model of lupus nephritis, the DNase I expression and protein concentration were decreased in kidneys, serum, and urine^57,58^. Similarly, in our study, the DNase activity in urine was lower in all animal models of AKI. Low DNase activity in urine could reflect decreased synthesis of DNase in the kidney and contribute to higher ecDNA in urine in some of the AKI animal models. However, further research is needed to analyse the DNase synthesis in various types of kidney injury and to follow the dynamics of the ecDNA. Moreover, administration of external DNase has proven protective against induced liver and kidney injury by our, as well as other groups^59,60^.

Indeed, our study has several limitations. Spearman’s rank-order test revealed some trends to correlation between clinical parameters specific for individual aetiologies and ecDNA, however none of them was significant due to low number of patients in some of the subgroups. Although patients were investigated the earliest after being diagnosed with AKI, their clinical state as to the time from the onset of symptoms, stage of AKI, and potential treatment before hospitalization might have differed. We neither investigated the origin of circulating nor urinary ecDNA. On the other hand, our cohort represents a reasonably large sample of children with AKI—population without comorbidities associated with increased ecDNA and/or changes in DNase activity. Moreover, we complemented the human study by animal experiments. These experimental results were obtained from adult males only to prevent variation caused by oestrous cycle. We are aware of the fact that this is a limitation and potential sex differences in i.e. DNase activity, could be relevant. Future studies should be performed to cover this issue.

In summary, our study suggests that an increase in ecDNA in AKI depends also on its aetiology. Even though the ecDNA represents a nonspecific marker, it seems to be increased in some types of renal injury, such as TIN and aHUS, already at the time of AKI diagnosis. Based on the pathophysiology of AKI induced in our models, we suppose that rise in urinary ecDNA reflects tubular injury. Further studies are needed to establish whether a rise in circulating and/or urinary ecDNA or its fractions or changes in the activity of DNase precede the clinical manifestation of AKI. Also, it is not clear yet, whether these could be used as prognostic markers in the case of disease manifestation. Knowledge of the origin of both circulating and urinary ecDNA in AKI of different aetiology, or size of the fragments could provide important diagnostic information. Knowledge of the role of inflammation and NETosis in the pathogenesis of AKI of different aetiology could provide a basis for potential therapeutic targets.

## Supplementary Information

Supplementary Tables.

Supplementary Figure S1.

Supplementary Legends.

## Data Availability

The raw data supporting the conclusions of this article will be made available by the corresponding author (Ľubomíra Tóthová) without undue reservation, under reasonable request.

## References

[1] Bellomo R, Kellum JA, Ronco C (2012). Acute kidney injury. Lancet.

[2] Hsu CW, Symons JM (2010). Acute kidney injury: Can we improve prognosis?. Pediatr. Nephrol..

[3] Pozzoli S, Simonini M, Manunta P (2018). Predicting acute kidney injury: Current status and future challenges. J. Nephrol..

[4] Rangaswamy D, Sud K (2018). Acute kidney injury and disease: Long-term consequences and management. Nephrology.

[5] Tuan PNH (2020). Serum and urine neutrophil gelatinase-associated lipocalin levels measured at admission predict progression to chronic kidney disease in sepsis-associated acute kidney injury patients. Dis. Mark..

[6] Gaião SM, de Paiva JAOC (2017). Biomarkers of renal recovery after acute kidney injury. Rev. Bras. Ter. Intensiva.

[7] Sexton EM, Fadrowski JJ, Pandian V, Sloand E, Brown KM (2020). Acute kidney injury in hospitalized pediatric patients: A Review of Research. J. Pediatr. Heal. Care.

[8] Li L (2016). Cell-free circulating mitochondrial DNA content and risk of hepatocellular carcinoma in patients with chronic HBV infection. Sci. Rep..

[9] O’Driscoll L (2007). Extracellular nucleic acids and their potential as diagnostic, prognostic and predictive biomarkers. Anticancer Res..

[10] Lo YMD, Han DSC, Jiang P, Chiu RWK (2021). Epigenetics, fragmentomics, and topology of cell-free DNA in liquid biopsies. Science.

[11] Celec P, Vlková B, Lauková L, Bábíčková J, Boor P (2018). Cell-free DNA: The role in pathophysiology and as a biomarker in kidney diseases. Expert Rev. Mol. Med..

[12] Poli C (2017). IL-26 Confers proinflammatory properties to extracellular DNA. J. Immunol..

[13] Tong S (2019). Role of neutrophil extracellular traps in chronic kidney injury induced by bisphenol-A. J. Endocrinol..

[14] Cao H (2019). Urinary mitochondrial DNA: A potential early biomarker of diabetic nephropathy. Diabetes Metab. Res. Rev..

[15] Truszewska A (2020). Cell-free DNA profiling in patients with lupus nephritis. Lupus.

[16] Clementi A (2016). The role of cell-free plasma DNA in critically ill patients with sepsis. Blood Purif..

[17] Merkle J, Daka A, Deppe AC, Wahlers T, Paunel-Görgülü A (2019). High levels of cell-free DNA accurately predict late acute kidney injury in patients after cardiac surgery. PLoS ONE.

[18] Oellerich M (2019). Absolute quantification of donor-derived cell-free DNA as a marker of rejection and graft injury in kidney transplantation: Results from a prospective observational study. Am. J. Transpl..

[19] Oellerich M (2021). Liquid biopsies: Donor-derived cell-free DNA for the detection of kidney allograft injury. Nat. Rev. Nephrol..

[20] Xie X (2019). Dialysate cell-free mitochondrial DNA fragments as a marker of intraperitoneal inflammation and peritoneal solute transport rate in peritoneal dialysis. BMC Nephrol..

[21] Bieber S, Muczynski KA, Lood C (2020). Neutrophil activation and neutrophil extracellular trap formation in dialysis patients. Kidney Med..

[22] Kim K (2021). Associations between cell-free mitochondrial DNA and inflammation, and their clinical implications for patients on hemodialysis: A prospective multicenter cohort study. Blood Purif..

[23] Homolová J (2020). Plasma concentrations of extracellular DNA in acute kidney injury. Diagnostics.

[24] Jansen MPB (2017). Release of extracellular DNA influences renal ischemia reperfusion injury by platelet activation and formation of neutrophil extracellular traps. Kidney Int..

[25] Okubo K (2018). Macrophage extracellular trap formation promoted by platelet activation is a key mediator of rhabdomyolysis-induced acute kidney injury. Nat. Med..

[26] Wang S (2018). DNase-1 treatment exerts protective effects in a rat model of intestinal ischemia-reperfusion injury. Sci. Rep..

[27] Akcan-Arikan A (2007). Modified RIFLE criteria in critically ill children with acute kidney injury. Kidney Int..

[28] Thomas ME (2015). The definition of acute kidney injury and its use in practice. Kidney Int..

[29] Schwartz GJ (2012). Improved equations estimating GFR in children with chronic kidney disease using an immunonephelometric determination of cystatin C. Kidney Int..

[30] Kovalčíková A (2018). Salivary creatinine and urea are higher in an experimental model of acute but not chronic renal disease. PLoS ONE.

[31] Dennhardt S (2018). Modeling hemolytic-uremic syndrome: In-depth characterization of distinct murine models reflecting different features of human disease. Front. Immunol..

[32] Čepcová D (2021). The protective effect of 1-methyltryptophan isomers in renal ischemia-reperfusion injury is not exclusively dependent on indolamine 2,3-dioxygenase inhibition. Biomed. Pharmacother..

[33] Badiou S, Dupuy AM, Descomps B, Cristolead JP (2003). Comparison between the enzymatic vitros assay for creatinine determination and three other methods adapted on the olympus analyzer. J. Clin. Lab. Anal..

[34] Geerdink LM (2012). Atypical hemolytic uremic syndrome in children: Complement mutations and clinical characteristics. Pediatr. Nephrol..

[35] Brocklebank V, Wood KM, Kavanagh D (2018). Thrombotic Microangiopathy and the Kidney. Clin. J. Am. Soc. Nephrol..

[36] Fuchs TA (2010). Extracellular DNA traps promote thrombosis. Proc. Natl. Acad. Sci..

[37] Fuchs TA, Kremer Hovinga JA, Schatzberg D, Wagner DD, Lämmle B (2012). Circulating DNA and myeloperoxidase indicate disease activity in patients with thrombotic microangiopathies. Blood.

[38] Ammollo CT, Semeraro F, Xu J, Esmon NL, Esmon CT (2011). Extracellular histones increase plasma thrombin generation by impairing thrombomodulin-dependent protein C activation. J. Thromb. Haemost..

[39] Fuchs TA, Bhandari AA, Wagner DD (2011). Histones induce rapid and profound thrombocytopenia in mice. Blood.

[40] Huang H (2011). Endogenous histones function as alarmins in sterile inflammatory liver injury through Toll-like receptor 9 in mice. Hepatology.

[41] Gloude NJ (2017). Circulating dsDNA, endothelial injury, and complement activation in thrombotic microangiopathy and GVHD. Blood.

[42] Sui J (2021). Plasma levels of S100A8/A9, histone/DNA complexes, and cell-free DNA predict adverse outcomes of immune thrombotic thrombocytopenic purpura. J. Thromb. Haemost..

[43] Psotka MA (2009). Shiga toxin 2 targets the murine renal collecting duct epithelium. Infect. Immun..

[44] Roy S, Awogbemi T, Holt RCL (2020). Acute tubulointerstitial nephritis in children– a retrospective case series in a UK tertiary paediatric centre. BMC Nephrol..

[45] Martinez Valenzuela L, Draibe J, Fulladosa X, Torras J (2020). New biomarkers in acute tubulointerstitial nephritis: A novel approach to a classic condition. Int. J. Mol. Sci..

[46] Munshi R, Hsu C, Himmelfarb J (2011). Advances in understanding ischemic acute kidney injury. BMC Med..

[47] Whitaker RM (2015). Urinary mitochondrial DNA is a biomarker of mitochondrial disruption and renal dysfunction in acute kidney injury. Kidney Int..

[48] Chang C-C (2019). Urinary cell-free mitochondrial and nuclear deoxyribonucleic acid correlates with the prognosis of chronic kidney diseases. BMC Nephrol..

[49] Zhang M (2021). Importance of urinary mitochondrial DNA in diagnosis and prognosis of kidney diseases. Mitochondrion.

[50] Kumar SVR (2015). Neutrophil extracellular trap-related extracellular histones cause vascular necrosis in severe GN. J. Am. Soc. Nephrol..

[51] Antonelou M, Evans RDR, Henderson SR, Salama AD (2020). Neutrophils are key mediators in crescentic glomerulonephritis and targets for new therapeutic approaches. Nephrol. Dial. Transpl..

[52] Kimura H, Mii A, Shoji J, Arakawa Y, Shimizu A (2021). Immunohistochemical detection of citrullinated histone H3-positive neutrophils is useful for identifying active glomerular and interstitial lesions in antineutrophil cytoplasmic antibody-associated vasculitis. Histopathology.

[53] Koizumi T (1995). Tissue distribution of deoxyribonuclease I (DNase I) activity level in mice and its sexual dimorphism. Exp. Anim..

[54] Koizumi T (1996). Genetic control of urinary deoxyribonuclease I (DNase I) activity levels in mice. Exp. Anim..

[55] Tsutsumi S (2001). DNase I is present in the chief cells of human and rat stomachs. Histochem. J..

[56] Basnakian AG, Ueda N, Kaushal GP, Mikhailova MV, Shah SV (2002). DNase I-like endonuclease in rat kidney cortex that is activated during ischemia/reperfusion injury. J. Am. Soc. Nephrol..

[57] Macanovic M, Lachmann PJ (1997). Measurement of deoxyribonuclease I (DNase) in the serum and urine of systemic lupus erythematosus (SLE)-prone NZB/NZW mice by a new radial enzyme diffusion assay. Clin. Exp. Immunol..

[58] Zykova SN, Tveita AA, Rekvig OP (2010). Renal Dnase1 enzyme activity and protein expression is selectively shut down in murine and human membranoproliferative lupus nephritis. PLoS ONE.

[59] Vokálová L (2017). Deoxyribonuclease partially ameliorates thioacetamide-induced hepatorenal injury. Am. J. Physiol. Gastrointest. Liver Physiol..

[60] Zhang H (2020). Hepatic surgical stress promotes systemic immunothrombosis that results in distant organ injury. Front. Immunol..

