# Supplementary material for: Extracellular DNA concentrations in various aetiologies of acute kidney injury
Source: Sci Rep. 2022 Oct 7;12:16812. doi: 10.1038/s41598-022-21248-7 (PMC9546839; doi:10.1038/s41598-022-21248-7)
Supplement: Supplementary file 1 — Supplementary Tables. [file 41598_2022_21248_MOESM1_ESM.docx]

**Supplementary material**

**Extracellular DNA concentrations in various aetiologies of acute kidney injury**

Alexandra Gaál Kovalčíková,^1, 2^ Ľubica Janovičová,^1^ Július Hodosy,^1, 3^ Janka Bábíčková,^1,4^ Diana Vavrincová-Yaghi,^5^ Peter Vavrinec,^5^ Peter Boor,^6^ Ľudmila Podracká,^2^ Katarína Šebeková,^1^ Peter Celec,^1, 7^ Ľubomíra Tóthová ^1^*

^1^ – Institute of Molecular Biomedicine, Faculty of Medicine, Comenius University, Bratislava, Slovakia

^2^ – Department of Paediatrics, National Institute of Children’s diseases and Faculty of Medicine, Comenius University in Bratislava, Bratislava, Slovakia

^3^ – Emergency Department Ruzinov, University Hospital Bratislava, Slovakia

^4^ – Department of Clinical Medicine, University of Bergen, Bergen, Norway

^5^ – Department of Pharmacology and Toxicology, Faculty of Pharmacy, Comenius University, Bratislava, Slovakia

^6^ – Institute of Pathology & Department of Nephrology, University Clinic of the RWTH Aachen, Germany

^7^ – Institute of Pathophysiology, Faculty of Medicine, Comenius University, Bratislava, Slovakia

***Corresponding author:**

Assoc. prof. Ľubomíra Tóthová, Dr., PhD

Institute of Molecular Biomedicine

Faculty of Medicine, Comenius University

Sasinkova 4, 811 08 Bratislava

Slovakia

[tothova.lubomira@gmail.com](mailto:tothova.lubomira@gmail.com)

Phone: +421 2 59357296

Fax: +421290119-631

| **Parameter** | **CTRL** | **AKI** |
| --- | --- | --- |
| **Number** | 27 | 28 |
| **Age (years)** | 8.6 ± 4.3 | 9.7 ± 6.3 |
| **Gender (F/M)** | 12/15 | 13/15 |
| **BMI (kg/m^2^)** | 18.7 ± 3.6 | 19.7 ± 4.8 |
| **Height (cm)** | 136 ± 26 | 135 ± 39 |
| **Creatinine (µmol/l)** | 47.4 ± 12.1 | 164.3 ± 98.5 |
| **BUN (mmol/l)** | 3.4 ± 0.6 | 13.5 ± 13.4 |
| **eGFR (ml/min/1.73m^2^)** | 107 ± 15 | 41 ± 23 |
| **Aetiology of AKI:** |  | GN (11%) |
|  |  | TIN (40%) |
|  |  | aHUS (30%) |
|  |  | Others (prerenal causes of AKI, small pelvic tumour, 19%) |

Supplementary Table S1: Characteristics of children with AKI and healthy controls

aHUS – atypical haemolytic uremic syndrome, AKI – acute kidney injury, BMI – body mass index, BUN – blood urea nitrogen, CTRL – healthy controls, eGFR – estimated glomerular filtration rate, F/M – females/males, GN – glomerulonephritis, TIN – tubulointerstitial nephritis. Data are expressed as mean ± SD

Supplementary Table S2: Relation between eGFR and extracellular DNA and its fractions in plasma and urine in children’s patients with AKI (regardless of aetiology).

|  |  | **Plasma ecDNA** | **Urinary ecDNA** | **Plasma ncDNA** | **Urinary ncDNA** | **Plasma mtDNA** | **Urinary mtDNA** |
| --- | --- | --- | --- | --- | --- | --- | --- |
| **eGFR** | r | -0.47 | -0.27 | -0.44 | -0.47 | -0.29 | -0.26 |
|  | p | ≤0.001 | =0.06 | ≤0.01 | ≤0.01 | =0.06 | >0.05 |

AKI – acute kidney injury, ecDNA – extracellular DNA, eGFR – estimated glomerular filtration rate, mtDNA – mitochondrial DNA, ncDNA – nuclear DNA, r – Spearman´s rank-order correlation coefficient. Values p ≤ 0.05 are considered as statistically significant (highlighted in grey).

Supplementary Table S3: Relation between clinical parameters and extracellular DNA and its fractions in plasma and urine in children’s patients with different aetiologies of AKI.

| **Aetiology** | **Clinical parameter** |  | **Plasma ecDNA** | **Urinary ecDNA** | **Plasma ncDNA** | **Urinary ncDNA** | **Plasma mtDNA** | **Urinary mtDNA** |
| --- | --- | --- | --- | --- | --- | --- | --- | --- |
| **GN** | **proteinuria** | r | 1.00 | 1.00 | 0.50 | 1.00 | 1.00 | 1.00 |
|  |  | p | >0.05 | >0.05 | >0.05 | >0.05 | >0.05 | >0.05 |
| **TIN** | **eGFR** | r | -0.47 | -0.18 | -0.07 | 0.17 | 0.31 | -0.30 |
|  |  | p | >0.05 | >0.05 | >0.05 | >0.05 | >0.05 | >0.05 |
| **aHUS** | **LDH** | r | 0.53 | 0.57 | 0.90 | 0.43 | 0.80 | 0.50 |
|  |  | p | >0.05 | >0.05 | >0.05 | >0.05 | >0.05 | >0.05 |
| **Others** | **eGFR** | r | 0.30 | 0.40 | 0.50 | 0.50 | -0.50 | 0.50 |
|  |  | p | >0.05 | >0.05 | >0.05 | >0.05 | >0.05 | >0.05 |

aHUS – atypical haemolytic uremic syndrome, AKI – acute kidney injury, ecDNA – extracellular DNA, eGFR – estimated glomerular filtration rate, GN – glomerulonephritis, LDH – lactate dehydrogenase, mtDNA – mitochondrial DNA, ncDNA – nuclear DNA, r – Spearman´s rank-order correlation coefficient, TIN – tubulointerstitial nephritis.

Supplementary Table S4: Relation between GFR and extracellular DNA and its fractions in plasma and urine in three different animal models of AKI.

| **Model** |  |  | **Plasma ecDNA** | **Urinary ecDNA** | **Plasma ncDNA** | **Urinary ncDNA** | **Plasma mtDNA** | **Urinary mtDNA** |
| --- | --- | --- | --- | --- | --- | --- | --- | --- |
| AN | **GFR** | r | -0.75 | -0.82 | -0.68 | -0.84 | -0.62 | -0.79 |
|  |  | p | ≤0.001 | ≤0.001 | ≤0.001 | ≤0.001 | ≤0.01 | ≤0.001 |
| HUS | **GFR** | r | -0.85 | -0.08 | -0.78 | 0.07 | -0.45 | 0.34 |
|  |  | p | ≤0.001 | >0.05 | ≤0.001 | >0.05 | =0.06 | >0.05 |
| IRI | **GFR** | r | -0.31 | -0.59 | -0.06 | -0.86 | 0.28 | -0.60 |
|  |  | p | ≤0.05 | ≤0.001 | >0.05 | ≤0.001 | >0.05 | ≤0.001 |

AKI – acute kidney injury, AN – adenine nephropathy, ecDNA – extracellular DNA, GFR – glomerular filtration rate, HUS – haemolytic uremic syndrome, IRI – ischemia-reperfusion injury, mtDNA – mitochondrial DNA, ncDNA – nuclear DNA, r – Spearman´s rank-order correlation coefficient. Values p ≤ 0.05 are considered as statistically significant (highlighted in grey).
