# Supplementary figures and images for: Extracellular DNA concentrations in various aetiologies of acute kidney injury
Source: Sci Rep. 2022 Oct 7;12:16812. doi: 10.1038/s41598-022-21248-7 (PMC9546839; doi:10.1038/s41598-022-21248-7)

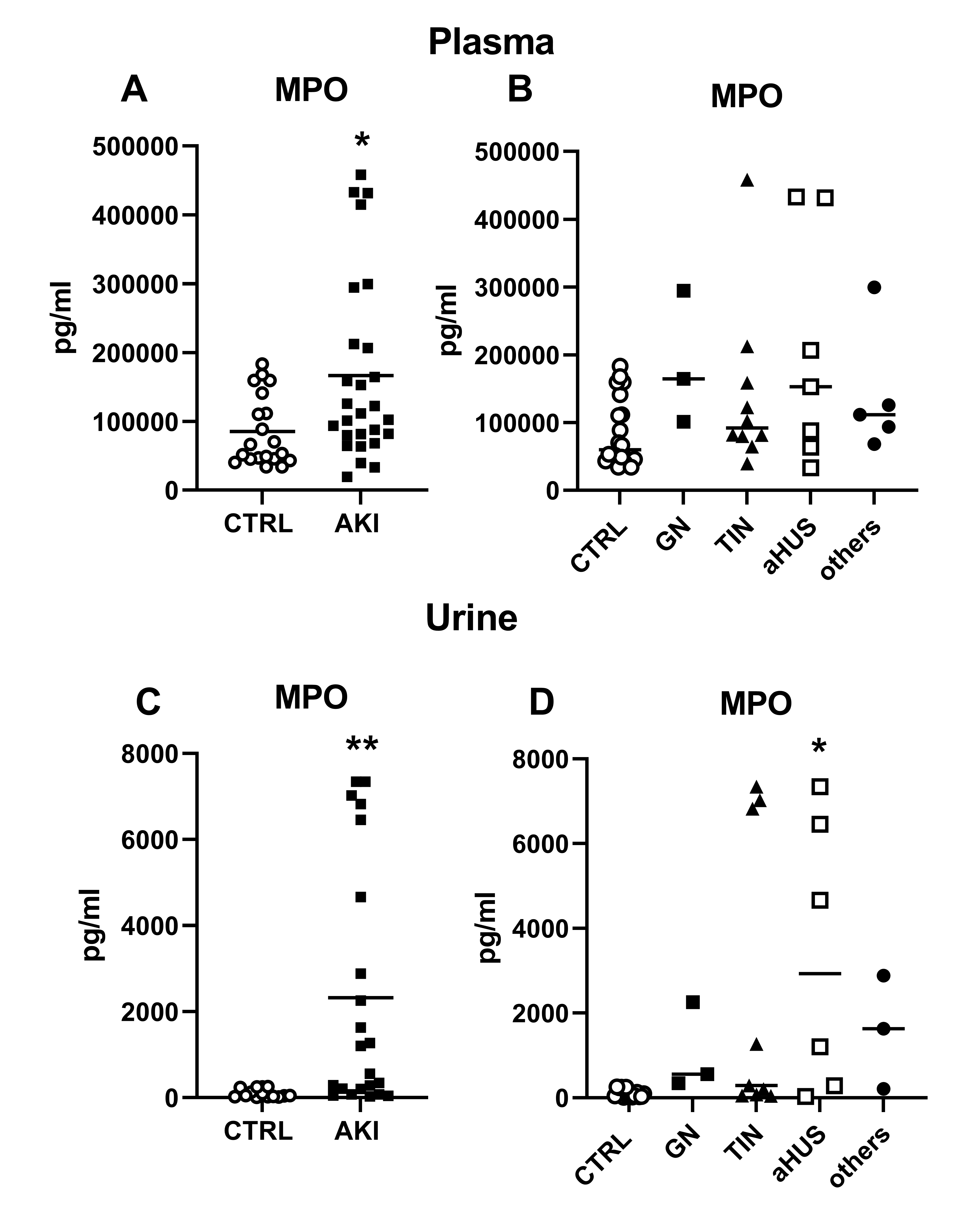

Supplement: Supplementary file 2 — Supplementary Figure S1. [file 41598_2022_21248_MOESM2_ESM.tif]
