# Supplementary material for: Extracellular DNA concentrations in various aetiologies of acute kidney injury
Source: Sci Rep. 2022 Oct 7;12:16812. doi: 10.1038/s41598-022-21248-7 (PMC9546839; doi:10.1038/s41598-022-21248-7)
Supplement: Supplementary file 3 — Supplementary Legends. [file 41598_2022_21248_MOESM3_ESM.docx]

**Supplementary Figure S1**: Concentrations of plasma and urinary MPO in children with AKI and healthy controls.

**A**: Plasma MPO in children with AKI regardless the aetiology and in healthy controls. **B**: plasma MPO in children with different aetiologies (subgroups) of AKI and healthy children. **C**: Urinary MPO in children with AKI regardless the aetiology and in healthy controls. **D**: urinary MPO in children with different aetiologies (subgroups) of AKI and healthy children.

aHUS – atypical haemolytic uremic syndrome, CTRL – healthy controls, GN – glomerulonephritis, MPO – myeloperoxidase, TIN – tubulointerstitial nephritis.
